# Rejoinder to McNeish and Mislevy: What Does Psychological Measurement Require?

**DOI:** 10.1007/s11336-024-10004-7

**Published:** 2024-10-30

**Authors:** Klaas Sijtsma, Jules L. Ellis, Denny Borsboom

**Affiliations:** 1 Department of Methodology and Statistics TSB, Tilburg University, PO Box 90153, 5000LE Tilburg, The Netherlands; 2 Open University of The Netherlands, Heerlen, The Netherlands; 3 University of Amsterdam, Amsterdam, The Netherlands

**Keywords:** classical test theory, factor analysis, item response theory, latent variable, sum score, theory development in psychology

## Abstract

In this rejoinder to McNeish (2024) and Mislevy (2024), who both responded to our focus article on the merits of the simple sum score (Sijtsma et al., 2024), we address several issues. Psychometrics education and in particular psychometricians’ outreach may help researchers to use IRT models as a precursor for the responsible use of the latent variable score and the sum score. Different methods used for test and questionnaire construction often do not produce highly different results, and when they do, this may be due to an unarticulated attribute theory generating noisy data. The sum score and transformations thereof, such as normalized test scores and percentiles, may help test practitioners and their clients to better communicate results. Latent variables prove important in more advanced applications such as equating and adaptive testing where they serve as technical tools rather than communication devices. Decisions based on test results are often binary or use a rather coarse ordering of scale levels, hence, do not require a high level of granularity (but nevertheless need to be precise). A gap exists between psychology and psychometrics which is growing deeper and wider, and that needs to be bridged. *Psychology* and *psychometrics* must work together to attain this goal.

Daniel McNeish (2024) and Robert Mislevy (2024) both responded to our article in *Psychometrika* (Sijtsma et al., 2024) on the status of the sum score in psychological measurement. We appreciate the effort they put in their interesting and thoughtful responses. Because the authors further illuminate the discussion and share their concerns with us and the readers of *Psychometrika*, we gladly accept the opportunity *Psychometrika*’s Editor granted us to publish this rejoinder. Because McNeish’s reaction is broader in content than Mislevy’s, much of our rejoinder focuses on his, but because both authors’ main issue seems to agree, at crucial points, our rejoinder concerns Mislevy’s comments as well. To avoid aimless nitpicking and because we already made our position clear in our focus article, we will not discuss all the detailed issues the discussants raise. Instead, we zoom in on the greater themes we think are important in present-day psychometrics. One theme concerns the importance of psychometrics education and in particular psychometricians’ outreach, which may help researchers to use IRT models as a precursor for the responsible use of the latent variable score and the sum score. Next, methods used for test and questionnaire construction, whether based on classical test theory (CTT) and factor analysis (FA) or item response theory (IRT), often do not produce highly different results, and when they do, this may be due to an unarticulated attribute theory generating noisy data. Another theme is that the sum score and transformations thereof, such as normalized test scores and percentiles, may help test practitioners and their clients to better communicate results. Latent variables prove important in more advanced applications such as equating and adaptive testing where they serve as technical tools rather than communication devices. Next, decisions based on test results are often binary or use a rather coarse ordering of scale levels, hence, do not require a high level of granularity (but nevertheless need to be precise). Finally, a gap exists between psychology and psychometrics which is growing deeper and wider, and that needs to be bridged. *Psychology* and *psychometrics* must work together to attain this goal.

## Back in Time?

In his response to our article, McNeish expresses concern that our discussion of the sum score may hamper the application of more advanced psychometric knowledge to test and questionnaire construction and the use of resulting instruments in research and psychological practice. Worse, he fears that lending the sum score credibility might “unintentionally preserve psychometric illiteracy among empirical researchers” (McNeish, 2024, p. 7), who lack psychometric training and thus are unable to value adequate psychometric analysis that must precede a decision to use the sum score. In addition, he claims that sum scores promote use of invalid measures, because “scale scores are frequently used without accompanying evidence that scores are necessarily meaningful (e.g., that they capture an intended construct or predict a relevant outcome)” (McNeish, 2024, p. 2).

Mislevy’s response is different from McNeish’s, but shares with his a distancing from the sum score perhaps most evident from the quote (Mislevy, 2024, p. 5) that “Sum scores tap into a deep intuition of quantity and that more good things is better, which to varying extents people share with apes, frogs, and guppies.” Without the context he provides, his quote could be interpreted just as well as a plea for the sum score, buying into a principle omnipresent in the natural world of which human beings are a part. But the wider context makes abundantly clear that he thinks the sum score must be considered part of what he calls intuitive test theory (ITT), where it serves a purpose at a low level of sophistication, for example, to provide a first impression of somebody’s performance level, but soon “Scientists and practitioners, as sophisticated as they may be in their own areas of expertise, in some fields a majority, gather and interpret assessment information using sum scores at the level of ITT, to the detriment of their applications (Mislevy, 2024).”

We think these quotes represent McNeish’s and Mislevy’s positions quite well. Both authors express the worry that even though the sum score may have desirable psychometric properties that can motivate its use in specific cases, our article may be read as an encouragement for researchers to continue with suboptimal psychometric approaches that have been described previously as “measurement by fiat” (Torgerson, 1958). Since neither of our discussants really questions the stochastic ordering and the reliability lower-bound results we discussed in our contribution, we will address the issue whether the use of the sum score as a scale for a psychological attribute takes us back in time and is damaging for the use of tests and questionnaires. What our discussants seem to say is that by discussing the fact that IRT models justify the use of the sum score, we are tempting researchers and test users to ignore more advanced IRT methods and fall back to simpler and especially more primitive methods. Of course, the discussants do not suggest this is the purpose of our article, but their concern seems to refer to what they expect is an unforeseen and unwanted side effect of our work. In this rejoinder, we try to take away the concern that the sum score takes us back in time.

## Education or Outreach?

Despite the provocative title of our article, we did not suggest that a defense of the sum score implies abandoning methods that employ latent variables, such as item response theory (IRT). What we posited was that, in an *ideal* psychometric world, the fact that an IRT model fits the data well can form an important justification for the use of the sum score. This follows from the results that a broad range of IRT models for dichotomously scored items implies the stochastic ordering property we discussed (i.e., stochastic ordering of the latent variable by the sum score (SOL); Sijtsma et al., 2024), whereas most IRT models for polytomous items justify the ordering property by approximation (Junker, 1991; Van der Ark, 2005). When McNeish suggests that empirical researchers lack psychometric training and thus are unable to value adequate psychometric analysis that must precede a decision to use the sum score, he implies that empirical researchers are unable to use IRT models in a responsible way. He is touching on a deeper problem here that deserves more attention.

Of course, we do not want to *encourage* researchers to use the sum score just like that and we did not *imply* they should do this either; that is, we do not mean to discourage more advanced approaches. The implication of our article was that, to use the sum score, one first has to establish the fit of an IRT model to the data (and even if that proves problematic, the sum score may still be a reasonable, predictive test score). But McNeish claims thoughtfully establishing IRT goodness-of-fit research is too much to expect from many empirical researchers, and expresses the worry that, given the absence of the relevant knowledge and skills, researchers may fall back to measurement by fiat   (Torgerson, 1958). He makes a very good point here. Compared to physicists, chemists, and biologists, psychologists are often less well trained in mathematics. They usually have received a few courses in basic statistics and psychometrics, and some researchers seek additional training in more advanced methods and software use that psychometricians provide. However, even though this may help to raise the level of statistics use in empirical research, the practice of statistics shows that many errors are made by researchers who do not seek advice from psychometricians at all or only after the data have been collected, and errors made at or before the phase of data collection cannot be corrected afterward (Campbell, 1974; Gardenier and Resnik, 2002; Hand, 2008, 2014; Kahneman, 2011). Software is continuously improved and made more user-friendly, but being able to start and execute a program is different from knowing precisely which decisions to make, the more so because real-data sets are much more complex than textbook examples show. Running a software program without knowing a lot of the statistics underlying it can cause great problems to the results of the research, as is constantly witnessed by the occurrence of questionable research practices and the replication crisis in psychology McNeish mentions (also, see Sijtsma, 2023).

Our position is this. To use the sum score responsibly, in many cases, one must first justify its use by demonstrating the fit of an IRT model. However, there are also situations in which a sum score can be useful, even when it is not supported by an IRT model, and the history and present-day practice of psychology show many examples. We will give further consideration to this topic in the next section. But first back to IRT. We agree with McNeish and notice that many researchers will not have knowledge advanced enough to bring an IRT analysis to a successful conclusion. More education may help but it is not as if thoughtful psychometrics education was never provided before: It was, but did not help enough. Therefore, most effectively, researchers will need the hands-on help of psychometricians. This requires an active attitude from both researchers who will have to ask for help and psychometricians who will have to reach out and provide help or advice. Empirical researchers could be trained to reach a higher level of knowledge about psychometrics, but this requires academic psychology training programs to make much more room for courses teaching them the much needed knowledge and skills. We have doubts whether this will happen, and know of examples where the program capacity for such topics was reduced rather than maintained, let alone increased. So, we thank McNeish for making the problem clear but also think he expects a bit too much of training programs and available courses and software.

## Test Construction

Is the situation in the practice of constructing psychological tests and questionnaires really so alarming as the previous section suggested? Before we delve deeper into this issue, we first notice that in our focus article, we considered the measurement of psychological attributes, such as cognitive abilities, personality traits, and attitudes more than educational measurement of knowledge and skills. In psychological measurement, the existence and use of well-established psychological theory about the attribute measured helps tremendously in giving direction to the test and questionnaire construction process (Sijtsma, 2012a). We sometimes have the impression that our discussants tend more than we do to educational measurement, which is all right, but the points of departure in educational test construction are concerned with educational goals, in particular concerning school subjects (e.g., arithmetic, language, history, geography) more than theoretical foundations of psychological attributes to be measured. This means that the question whether the measurement represents the attribute well in psychology is replaced with the question whether the item set represents the pool of possible items well (e.g., fraction arithmetic, English language vocabulary, history of the Weimar Republic, geography of Latin America). Simply put, construct validity in psychology is contrasted with content validity in education, and these are different measurement issues. Of course, there are also many similarities, perhaps more than there are differences, but it is useful to make the distinction because it might sometimes explain different viewpoints. In the next section, we will nevertheless (also) draw extensively from educational measurement when discussing use of latent variables and alternative scoring methods.

McNeish refers to the work of the COTAN (Evers, Sijtsma, Meijer, & Lucassen, 2010; also, see Sijtsma, 2012b), a committee working under the auspices of the Netherlands Institute for Psychologists (https://psynip.nl; unfortunately, the website only has a Dutch-language version; but see Evers, Lucassen, Meijer, & Sijtsma, 2009, 2015), responsible for the quality assessment of psychological tests and questionnaires used in the Netherlands and the Dutch-language part of Belgium. Based on the COTAN’s data base, it is clear that most tests are constructed using available (but not necessarily well established) theory of the attribute under consideration. Data collected and used by the test constructor for assembling their test or questionnaire are usually submitted to a principal component analysis (PCA) or a factor analysis (FA) to assess whether the dimensionality of the item set used is consistent with the test constructor’s intentions. These methods are also used to assess whether individual items must be reformulated or removed from the item set. Assessment is usually done using the loadings of items on factors resulting from rotation. The loadings provide the strength of the relation of an item with a weighted average of the item scores defining the factor score. It is quite rare that factor scores (i.e., latent variable scores in FA) are used eventually for scoring individuals on the test or questionnaire, and test constructors and test users often resort to the sum score or a transformation thereof, such as a normalized score or a percentile score. After a decision is made of the final composition of (sub) tests or (sub) questionnaires, one of the methods for estimating the reliability of the sum score or a transformation of the sum score is used. Almost always, coefficient alpha (Cronbach, 1951) is used for this purpose (see Oosterwijk, Van der Ark, & Sijtsma, 2019, for COTAN results).

Gradually, over the past one or two decades, several test constructors have started using IRT as well for test construction. The advantage of IRT over PCA and FA is that it is suited for discrete variables, whereas PCA and FA are suited for continuous variables. PCA and FA applications often circumvent the discreteness of the item scores by using tetrachoric (in case of dichotomously scored items) or polychoric (in case of discrete ordered rating-scale scores) inter-item correlations. These correlations assume that the item scores originated from the discretization of normally distributed, hence continuous item scores that are hypothetical in the sense that they were never observed but instead discretized to produce observable item scores. Several authors (e.g., Kappenburg-Ten Holt, 2014; Takane & De Leeuw, 1987) have pointed out the relationship between FA models and normal-ogive IRT models. We will leave discussions about similarities and differences between models for what they are and notice that IRT models are explicitly designed for discrete item scores and do not have to assume normality of latent item scores. Be that as it may, we notice that if attributes are well defined and operationalized, and one expects a unidimensional scale, then, because the point of departure is so strong, it may not matter a lot which model one used for assessing the item set because all models will probably produce (nearly) the same selections. Of course, there will be situations in which using one or the other model makes a difference for the final item selections, but the difference may not be overwhelming. However, when it is, making a choice is not easy. In particular, one may question why the choice of the method is so important for the final result when the methods are not worlds apart and the operationalization of the attribute leaves little room for highly different results. However, if the attribute theory is inarticulate, the structure in the data may be noisy and differences between methods may be the result of method peculiarities making it difficult to underpin the preference of one method over the other.

Both McNeish’s claim implicit in criticizing CTT and the sum score for its alleged simplicity and Mislevy’s characterization of the sum score as intuitive seem to imply that users of IRT think better and deeper about their measurement instruments than researchers using PCA and FA (and CTT reliability and CTT-related methods), which is an implication we suspect is difficult to substantiate. It may be true—and we will not dispute the literature results McNeish quotes—that researchers using sum scores often do not report validity results, but is it also true that researchers using IRT models and latent variables *do* abundantly report validity results? Or do these researchers mistake the use of an IRT model with a sound construct validity study? We think one cannot rule out that adherents of the “classical” approach of PCA and FA (and CTT methods) and their IRT-using colleagues both spend equal amounts of energy in designing their measurement instruments, but simply use different psychometric methods to attain their goals.

Importantly, however, we do agree with our discussants that IRT models make assumptions about *hypothetical* response processes explicit, likely more than PCA and FA do, but we also think that such assumptions are often limited, even rather superficial. For example, IRT models assume that the probability of a positive response is a monotone function of the scale for the attribute, but that is a rather modest, even intuitive, hence superficial specification not providing us with much insight into the precise response process (but see De Boeck & Wilson, 2004, for more ambitious modeling attempts). The monotonicity assumption is absent in PCA and FA, rendering IRT a somewhat greater potential for improving theoretical understanding of measurement. If our discussants’ preference for using IRT and latent variables is driven by a desire to urge researchers and test constructors to think hard about their measurement efforts, we can only concur. But again, we find it difficult, even implausible, to imply that researchers using other methods do not make similar efforts, even if PCA and FA are not as explicit about the response processes. One might as well posit that the absence of insightful assumptions about the response process in these models urges the researcher to think harder about the validity of their measurements, because she cannot fall back on a model that includes assumptions referring to the response process (which still do not relieve the researcher from her task to think hard about validity issues not implied by any model). In the next section, we discuss our position with respect to the latent variable and the sum score.

## Latent Variable Scores and Sum Scores

To abundantly make clear our position concerning latent variables: we simply like them, just as we like the sum score. Here is an explanation for our dual act of sympathy.

Our focus article referred to the stand-alone test or questionnaire used for psychological measurement, a fixed set of items (we limit the discussion to items as in arithmetic problems and rating-scale personality self-assessment statements or attitude statements) administered to all participants in a research study and all individuals in a diagnostic assessment session. Everybody responds to the same items. In this situation, the SOL result we discussed in our article is correct (for polytomous items SOL is correct by approximation, except the partial credit model for which it simply is correct), provided of course that the typical IRT assumptions necessary for deriving the SOL result are valid for the data at hand. Thus, if the IRT model fits the data, both the use of the latent variable score and the use of the sum score can be justified. The latter addition seems to be necessary, because experience shows that IRT models usually do not fit the data well when the models’ assumptions are seriously tested (Sijtsma and Van der Ark, 2021). But if we allow some discrepancy between model and data, if only for practical purposes, then both the latent variable estimate and sum score are justified.

We offer a few remarks about interpretation and communication value (see also Hemker, 2023). We focus on educational measurement, but the discussion applies to psychological measurement as well. The latent variable score has the problem that its values seem odd to non-experts. Being told that your son has a score of $-0.64$ for arithmetic may elicit some wild thoughts that can be prevented by simply transforming the scale to something that feels more familiar and acceptable. Cito, the well-known educational test provider in The Netherlands, decided to transform^1^ the scale of one of their famous advisory tests for secondary education to a scale ranging from 501 to 550, and report percentiles as well. This choice of scale range was completely arbitrary, not only avoiding technicalities resulting from the IRT scale confusing receivers of test results, but also avoiding the Dutch classic rating system (De Groot, 1966) running from 1 to 10 with 6 as the cutoff between insufficient (score 5) and sufficient (score 6) performance. They did this to evade too great familiarity often tempting laypeople to assign absolute meaning to numbers.

A more principled reason not to use latent variable scores in the practical reporting of test performance is that latent variable scores are based on the implicit (i.e., unknown to the examinee) differential weighing of the items in the test resulting from the maximum likelihood estimation procedure (and other estimation procedures as well) without informing the examinee prior to the testing. This could be avoided by pre-testing (on a training sample), estimating the items’ discrimination parameters, and then letting the examinees know which items are more important than others prior to being tested. However, this information could change the item parameters when new examinees who are well-informed about different item weights from pre-testing are tested, and these weights would become invalid. It seems we find ourselves in a dilemma. In smaller-scale applications, there is no pre-testing and we do not know how the items will be weighed, so that informing examinees prior to testing would be impossible. But if weights would be available, either in larger- or smaller-scale testing programs, imagine the task one would be faced with explaining prior to testing why there is differential weighting and based on what. Given that weights are difficult to predict and result from statistical estimation, we expect weighting would elicit serious and understandable protests and resistance, predictably referring to lack of transparency and test fairness. Such objections are difficult to handle let alone to defend. Equal item weighting at least has the advantage of simplicity, not raising doubt and resistance, while a larger number of items might compensate for a possible loss of measurement precision if not the statistically optimal weights are used. Thus, the sum score is intrinsically more transparent than the latent variable score, which is an important advantage (De Groot, 1970).

We now return to more technical issues, especially, the usefulness of latent variables in psychometric applications when testing is not stand-alone. This concerns the use of different test versions for the same attribute in different groups or the same group when tested repeatedly at different occasions. Different groups can have different ability levels, requiring items having different difficulty levels. The same group can be tested repeatedly on different occasions, requiring different items to avoid memory effects or anticipating a changing level between occasions. Different groups may also be tested at different occasions but for the same purpose, for example, when different cohorts of students are administered a final school exam that must be replaced with each next cohort to avoid items from becoming known, while the exams’ levels must remain the same for comparison and test fairness. This example refers to the practice of national testing at high schools in The Netherlands, where the requirement of comparability is politically motivated. In each example, performance on different tests must be made comparable across test versions. IRT and its latent variable have proven to be suited for the job, tolerating less than perfect but acceptable model fit. As an aside, we notice that also for CTT and test scores such as the sum score equating methods are available. Adaptive testing, where the items presented to an individual are tailored to her level as estimated based on the items she already responded to, thus gaining measurement precision using relatively few items, is another example where IRT and the latent variable are valuable tools.

## Score Granularity

Assuming the existence of a scale for a theoretically and empirically well-founded attribute, McNeish points out that the sum score only distinguishes a limited number of discrete scale scores, whereas the latent variable score enables an almost infinite number of values, thus approximating continuity, and considers this an advantage. He points out that SOL links each sum score to an expected value of the latent variable, and considers this a serious loss of information. Ignoring the fact that SOL refers to the ordering of complementary cumulative distributions (Sijtsma et al., 2024) of which expected values represent only one feature, the key question we address here is whether discreteness damages the practice of test use and when it does not, whether continuity serves any additional purpose and if so, what that purpose is.

First, we notice that, in many cases, psychological measurement results in noisy data that may reflect a sizeable amount of random measurement error, even when tests have high reliability. In practice, it is almost impossible to significantly distinguish two adjacent sum scores, let alone that one would be able to distinguish (say, standard normal) latent variable values of 1.23 and 1.24 or 1.26. One needs extremely high measurement precision (not necessarily high reliability; Emons, 2023; Mellenbergh, 1996) to realize such distinctions, meaning that confidence intervals for true scores and latent variables need to be extremely narrow, thus requiring very small standard errors. This usually requires a very long test or questionnaire, unattainable in practice for reasons of efficiency (e.g., available testing time, fatigue, learning and memory, running out of credible items).

Second, apart from random measurement error, a practical question is how much precision psychological measurement and its applications actually require. Our answer is: In many cases, much less than psychometrics can provide. The reason for this is that such advanced uses match neither the underlying theory, which is typically verbally stated and imprecise, nor the application, which often only requires ordering. Certainly, if psychometrics’ task was computing how to launch a spacecraft to the Moon to have it land on the right spot without endangering the astronauts, extreme precision would be mandatory. But decisions made with tests and questionnaires, although practically important without hesitation, are not as fine-grained as this. Perhaps the lower granularity of the decisions needed justifies discrete scales.

Third, if we take a closer look at the practice of psychological testing, this helps us to understand that a psychologist testing an individual client or a patient usually works in an environment without psychometric support and fully depends on the test manual plus scoring instruction, available physically or computerized. In this situation, it is difficult if not impossible to estimate a latent variable score, but a score count and possibly a transformation table to normed scores in whatever form desirable is very helpful and realistic. Moreover, it is important to realize that most decisions using tests and questionnaires entail classifying tested people in a few categories, often only binary: succeed—fail (education), hire—reject (jobs), and admit—not admit (therapy), but less coarse classifications are also required, such as the diagnosis of intelligence in a number of increasing levels and the assessment of change due to practice (education) and therapy (clinic). What is sometimes absent in discussions among psychometricians, is that in practice, decisions are hardly ever based on one test score but use other information sources as well (e.g., for a job applicant, additional test scores, education, vita, relevant job experience, assessment, references, committee advice; and for a student, dozens of test results, papers, skills trainings, group discussions). The optimal combination of these sources is a cause for concern, but we refrain from this highly relevant topic (but see Dawes, 1979; Meijer, Niessen, & Neumann, 2023; Kahneman, Sibony, & Sunstein, 2021).

The situation can be completely different in large-scale educational testing where thousands of students are tested within a short period of time, and a professional organization behind the testing provides the psychometric know-how for latent variable estimation with relatively good precision, sometimes even enabling the re-assessment or deletion of items that did not work out as expected. Often, for the purpose of communication such estimates are transformed to scales that are better comprehensible and communicable, as we discussed previously. We emphasize once more that our work focuses on psychological tests and psychological testing, which usually take place in one-to-one meetings as part of a more comprehensive professional consultation aimed at reaching a decision about a student, an applicant, or a patient with respect to their future well-being.

Finally, McNeish suggests that machine learning and other methodologies, for example, focusing on individual items thereby surpassing the conceptual foundation of measurement, may prove better methodologies for prediction. This is a position that can certainly be defended, and that sits well with the general maxim that measurement and prediction are often orthogonal activities; sometimes, improving prediction may harm measurement and vice versa. The study of the relation between measurement and prediction is, in our view, in need of further investigation given the rapid advance of atheoretical methods based on data science and artificial intelligence. The future will tell whether data-based approaches have merit in the context of measurement as well as prediction.

## Discussion: Back to the Future

Psychometrics education and in particular psychometricians’ outreach may help researchers to use IRT models as a precursor for the responsible use of the latent variable score and the sum score alike, and this is true even when some psychometricians do not like the sum score and rather prefer the latent variable. In this respect, it is important to distinguish the use of the latent variable *model* and the use of the latent variable *score*: the fit of a latent variable model can be an important justification for the use and interpretation of test scores, but that does not imply that the latent variable score should invariably be used. In fact, in many cases the sum score can be preferable for non-psychometric reasons such as simplicity and transparency (De Groot, 1970).

As we have argued, the sum score and transformations thereof, such as normalized test scores and percentiles, may help test practitioners and their clients to better communicate results. Test scores based on differential item weighting, either resulting from statistical estimation procedures or from deliberate *non-statistical* (but rather substantive) choices to weight one type of problem heavier than another, require deep explanation that examinees and clients may find impossible to understand and will experience as unfair. Moreover, such explanations may be unavailable because they were unanticipated, but simply result from a statistical estimation procedure that takes full advantage of the data using a mathematical criterion without substantive basis. Decisions based on test results are often binary or use a rather coarse ordering of scale levels, hence, do not require a high level of granularity (but nevertheless need to be precise). Prediction of criterion behavior such as education, job, and therapy success based on individual item scores such as McNeish suggests rather than total scores that represent a psychological attribute or a domain of knowledge and demarcated skills, easily runs into communication problems, most likely because it lacks a substantively interpretable foundation.

The problem perhaps most pervasive in psychological measurement is that *psychology* in general, exceptions noted, has shown too little interest in the painstaking endeavor of theory development for attributes that might better support the construction of valid tests and questionnaires. Many authors have discussed the weak substantive foundation of psychological measurement (e.g., Briggs, 2002; Michell, 1999; Sijtsma & Van der Ark, 2021) as well as the inarticulate nature of psychological theory (Fried, 2020; Oberauer and Lewandowsky, 2019). The current wave of theory building and formal modeling that arose in the wake of the replication crisis is encouraging (e.g., see Borsboom et al., 2021; Proulx & Morey, 2021; Robinaugh et al., 2024; Van Dongen et al., 2024; Van Rooij & Baggio, 2021). Meanwhile, psychometrics has made much progress, but the progress is technological more than based on new insights regarding attribute measurement, simply because psychology does not or hardly does supply these insights. Many authors (Borsboom, 2006; Borsboom et al., 2004; Markus and Borsboom, 2013; Sijtsma, 2009, 2012a, b) have warned that a gap exists between psychology and psychometrics which is growing deeper and wider as time goes by. The gap needs to be bridged. This must become a major point of discussion and concern, in *psychology* but also among *psychometricians* who are now too much focused on their models and too little on what they must measure.

## Footnotes

1 This transformation was more complex than simply (linearly) transforming a (latent variable) scale to another test score scale by using one formula. Because our discussion serves to clarify that latent variable scores are often non communicative and not how Cito transformed their scale, we refrain from further discussion of the exact transformation. We thank Bas Hemker for providing the information.
